# Initial collection, characterization, and storage of tuatara (*Sphenodon punctatus*) sperm offers insight into their unique reproductive system

**DOI:** 10.1371/journal.pone.0253628

**Published:** 2021-07-08

**Authors:** Sarah K. Lamar, Nicola J. Nelson, Jennifer A. Moore, Helen R. Taylor, Susan N. Keall, Diane K. Ormsby

**Affiliations:** 1 Centre for Biodiversity and Restoration Ecology, School of Biological Sciences, Victoria University of Wellington, Wellington, New Zealand; 2 Biology Department, Grand Valley State University, Allendale, Michigan, United States of America; 3 Royal Zoological Society of Scotland, Edinburgh, United Kingdom; 4 Department of Anatomy, University of Otago, Dunedin, New Zealand; 5 School of Biological Sciences, Victoria University of Wellington, Wellington, New Zealand; University Hospital of Münster, GERMANY

## Abstract

Successful reproduction is critical to the persistence of at-risk species; however, reproductive characteristics are understudied in many wild species. New Zealand’s endemic tuatara (*Sphenodon punctatus)*, the sole surviving member of the reptile order Rhynchocephalia, is restricted to 10% of its historic range. To complement ongoing conservation efforts, we collected and characterized mature sperm from male tuatara for the first time. Semen collected both during mating and from urine after courting contained motile sperm and had the potential for a very high percentage of viable sperm cells (98%). Scanning electron microscopy revealed a filiform sperm cell with distinct divisions: head, midpiece, tail, and reduced end piece. Finally, our initial curvilinear velocity estimates for tuatara sperm are 2–4 times faster than any previously studied reptile. Further work is needed to examine these trends at a larger scale; however, this research provides valuable information regarding reproduction in this basal reptile.

## Introduction

The persistence of viable populations of at-risk species hinges on them reproducing successfully. Thus, understanding the reproductive ecology and physiology of wild animals is essential for effective conservation. Often this basic information is limited or lacking for wild animals, with much of our understanding derived from domesticated species like cattle or laboratory species like mice [1, 2]. Further, limited investigations into the sperm characteristics of wild species have shown high variability, even between closely related species [3–6]. This reduces our ability to extrapolate inferences from domestic to non-domestic species and warrants considerable further research on the reproductive characteristics of wild animals.

A number of reproductive techniques, including non-invasive hormone monitoring, artificial insemination, and sperm banking, have had positive outcomes for threatened and endangered species that have experienced severe demographic declines [7, 8]. Although assisted reproductive techniques (ARTs) are never preferred over natural reproduction in large, self-sustaining populations, they can provide 1) breeding programs with a source of captive-bred individuals for reintroductions to the wild, 2) enhanced opportunities for genetic management of inbred populations, and 3) genetic banking of materials (i.e., gametes) to ensure future persistence [1]. Furthermore, moving semen for artificial insemination of endangered species is often logistically easier than moving individual animals. For example, an unmanned drone was used to transport semen from endangered kākāpō (*Strigops habroptilus*) across the difficult terrain of New Zealand’s Codfish Island/Whenua Hou [9, 10]. Conservationists were able to use artificial insemination to manage genetic diversity and increase fertility rates of these critically endangered birds without adversely affecting the survival of individuals [9].

**Fig 1:**
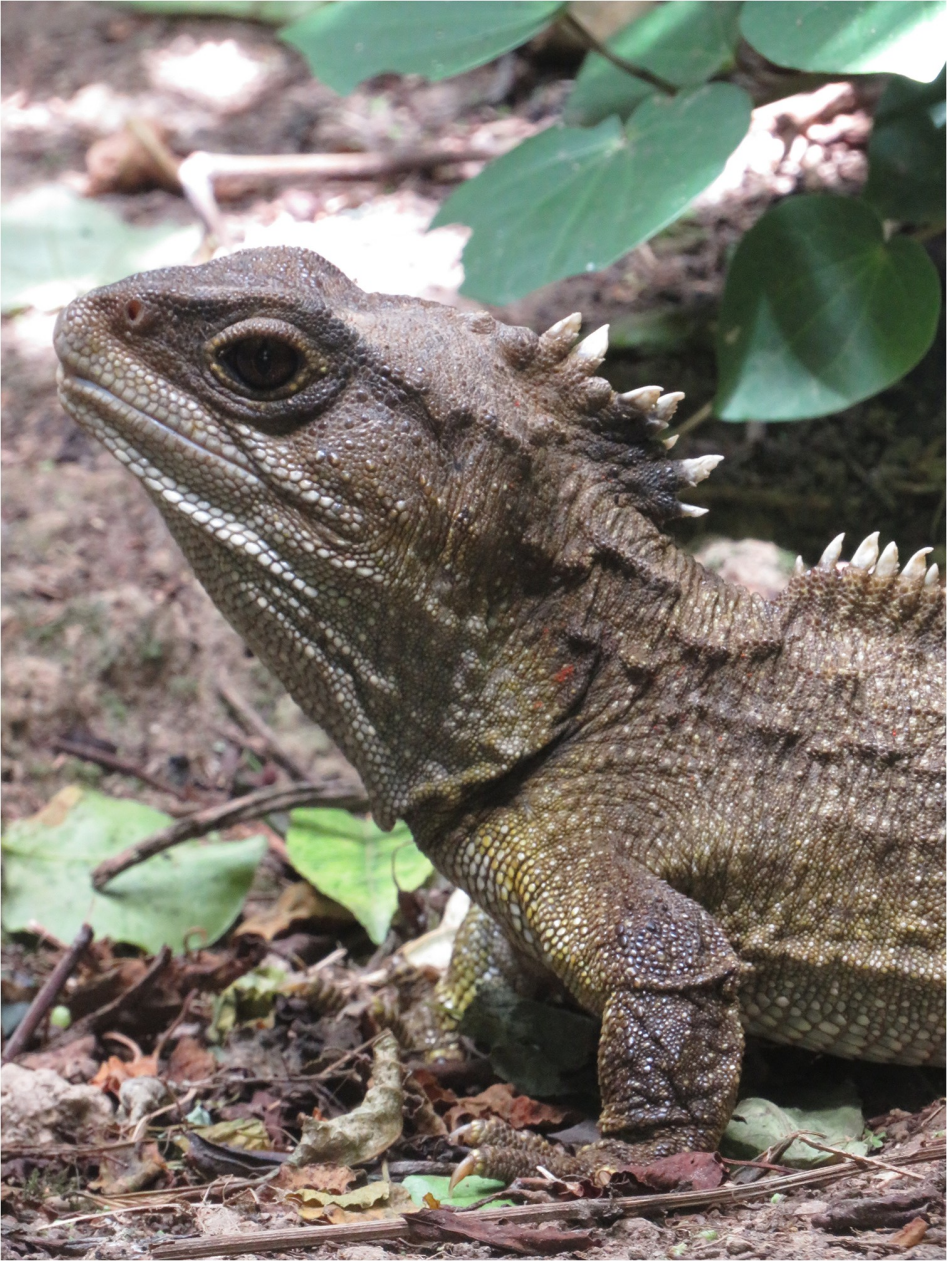
A male tuatara (*Sphenodon punctatus*) stands outside his burrow. Tuatara are sexually dimorphic at maturity, with males being on average larger, having a wider jaw, more triangular head, and a crest with larger, more closely arranged spikes.

Another species of conservation importance that could benefit from ARTs is the tuatara (*Sphenodon punctatus*) (Fig 1), a reptile endemic to New Zealand and the only surviving member of a once widespread order, the Rhynchocephalia [11]. Extreme distributional and demographic declines of tuatara populations prompted a massive conservation effort by the early-1990s [12]. While numerous conservation techniques have proven effective at restoring tuatara populations, such as mammal eradications, translocations, and headstarting programs [13–17], there is still much work to be done, including around ARTs.

The ability to collect and preserve semen for artificial insemination in wild tuatara has two major potential benefits for their conservation. First, tuatara are currently distributed across isolated island sites and a few fenced mainland ecosanctuaries. If gene flow between these disparate sites was deemed necessary, this could be initiated using ARTs instead of individual translocations, with potential benefits for animal welfare and reduced risks of disease transmission. Secondly, the movement of tuatara outside New Zealand is heavily restricted and they do not appear to readily breed in captivity. While six zoos outside New Zealand hold tuatara, only one has ever produced offspring (pers. comm. Simon Eyre). Thus, artificial insemination may be necessary to ensure the continued existence of captive tuatara outside their native range.

To date, semen has never been successfully collected from wild tuatara, and the only characterization of sperm from this species was conducted primarily using a single formalin-fixed testicular sample [18]. This work indicated that sperm were filiform with a total length ranging from 140–144 μm [18]. The acrosome and head formed a helically-shaped head region roughly 58–60 μm long [18]. The midpiece and tail were 7–8 μm and 74–78 μm long, respectively [18]. Finally, a short (2–4 μm) end piece was observed [18]. This study also found many internal features of the sperm cell considered to be plesiomorphies with Crocodilia and Testudines [18]. However, there were no mutual apomorphies characteristic of the order of reptiles most closely related to Rhynchocephalia, the Squamates [18, 19]. While these results serve as a starting point for current research, samples collected from the testes have not undergone maturation during their passage through the epididymis, which allows the sperm cell to develop the ability to fertilize a female ovum [20, 21]. Post-testicular sperm cell maturation was initially thought to only occur within Mammalia; however, research conducted on the saltwater crocodile (*Crocodylus porosus*) [22] and broad banded water snake (*Nerodia fasciata*) [23] has confirmed that this phenomenon is present in some reptile species. In light of these developments, the need to characterize sperm that has passed through the reproductive tract of the male tuatara is clear. Because tuatara are a basal lineage, their sperm characteristics may differ considerably from other reptiles [19].

**Fig 2:**
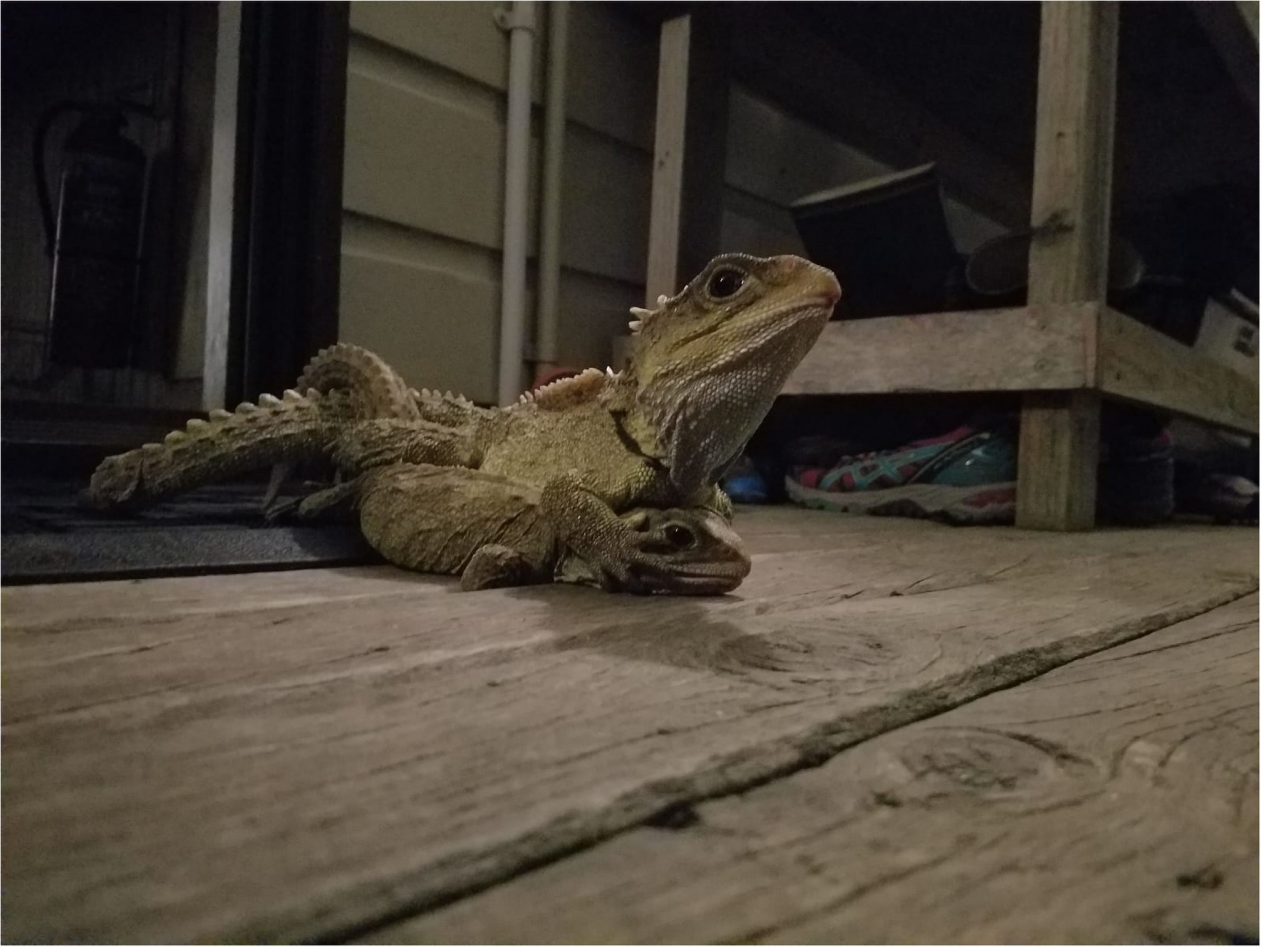
Tuatara (*Sphenodon punctatus)* mating. Tuatara mating on the porch of a Department of Conservation ranger house on Takapourewa. The male is postured on top of the female, with his hind limbs and tail twisting to oppose their cloaca. Tuatara are unique among reptiles for their lack of intromittent organ.

Research investigating sperm collection and the effects of diluents and cryoprotectants on sperm are extremely limited among reptiles, so much of our understanding of sperm science comes from birds. Electroejaculation, cloacal lavage, and digital massage are techniques used commonly in sperm studies. While electroejaculation has been used successfully in several bird [24, 25] and reptile [26, 27] species, it requires anesthesia and frequently results in samples heavily contaminated with urine [28, 29]. Further, tuatara are unique among reptiles for their absence of intromittent organ, with sperm transfer taking place via cloacal apposition [30] (Fig 2), making electrostimulation impossible in this species. Sperm collection via cloacal massage is well established in passerine birds and occurs by gently massaging the cloacal protuberance where sperm is stored [31, 32]; this method can also be adapted for bird species with penis-like copulatory appendages [28]. In reptiles, sperm collection via cloacal massage has been successfully performed on live, captive corn snakes (*Elaphe guttata*) [33] and the McCann’s skink (*Oligosoma maccanni*) [34]. Sperm has also been collected by massaging the terminal segment of the ductus deferens of captive Australian saltwater crocodiles (*C*. *porosus*) [35]. In both the saltwater crocodile [36] and the Argentine boa (*Boa constrictor occidentalis*) [37] massaging and/or probing the base of the hemipenis has led to successful sperm collection. However, the lack of an intromittent organ in tuatara again renders sperm collection via this method unfeasible [38]. Thus, developing a reliable method of collecting live sperm samples from this species is needed to progress any kind of male reproductive study.

After collection, effective storage of sperm is required to keep samples viable for ART use. Sperm are stored in a combination of diluents and/or cryopreservatives, depending on the desired storage period. Diluents are selected to optimize the retention of sperm motility and viability and are frequently used in ART when semen samples have small volumes associated with high sperm concentrations. While relatively little work has been done on diluent and cryoprotectant use in reptiles, initial results indicate it may be possible to maintain viable sperm at low temperatures without cryopreservation for several days [27, 29]. However, cryopreservation may be required for transferring semen between disparate, remote populations, where travel between sites can be difficult and weather-dependent. Cryoprotectants, along with careful freezing protocols, are used to minimize ice damage and ensure adequate cell dehydration; however, osmotic tolerances to freezing and cryoprotectants are species-specific and often require extensive testing, and many of the mechanisms governing the efficacy of cryoprotectants in different species remain unknown [2, 39].

In summary, sperm are an extremely variable cell type that can have disparate reactions to collection and storage protocols, even among closely related species. Having a thorough understanding of the morphology and movement patterns of mature sperm collected from adult tuatara will not only improve our understanding of tuatara mating systems, but also allow us to begin to develop storage protocols for use in genetic management and ARTs. In light of the evolutionary and conservation importance of the tuatara, we undertook this study with three aims: 1) to develop a method for semen collection from wild tuatara, 2) to describe mature tuatara sperm for the first time, and 3) to assess the success of various storage buffers, both diluents and cryoprotectants, for tuatara semen.

## Materials and methods

### Sample collection

This research was permitted by Grand Valley State University’s Institutional Animal Care and Use Committee (permit #19-12-A), Victoria University of Wellington’s Animal Ethics Committee (permit #27041), and by the Department of Conservation (permit # FAU 50568) in consultation with Ngāti Koata.

From 26 Feb– 3 Mar 2019, we captured wild tuatara on Stephens Island/Takapourewa (located in the Marlborough Sounds, New Zealand). Takapourewa harbors the largest extant population of tuatara, and is the only wild population for which there is information on the mating system [40, 41] and reproductive cycles [42] of these animals. Mating corresponds with peak male testosterone concentrations, which occur during austral late-summer to early autumn (Feb-March) [42]. We targeted males that were actively engaged in behavioral interactions with other tuatara. Interactions included male-male aggression, courtship, and mating [43].

**Fig 3:**
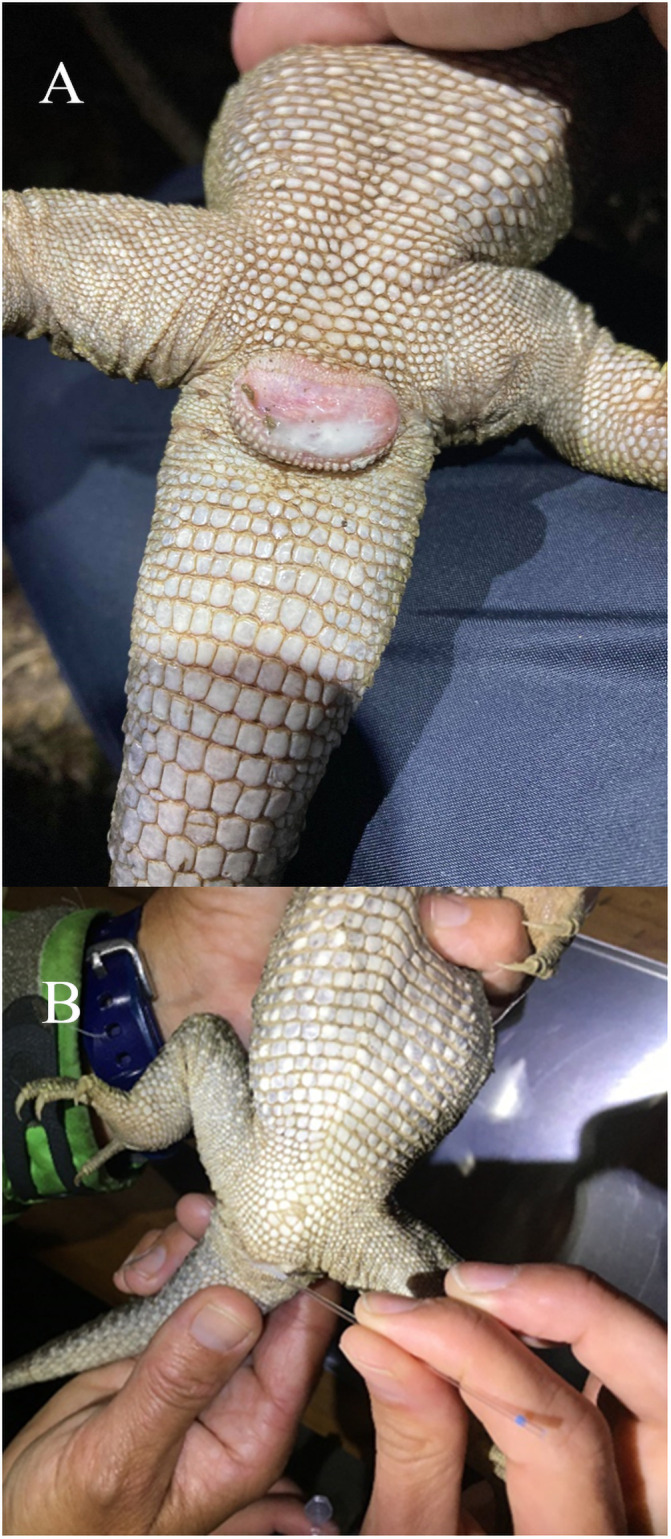
Sperm visible on a female’s cloaca after mating and collection. (A) Sperm is visible on the female’s cloaca after separating a mating pair. (B) Sperm being collected from the female’s cloaca with a microcapillary tube.

We trialed several different methods of collecting semen from tuatara. We first attempted to obtain sperm by cloacal lavage. We flushed the cloaca with either phosphate buffered saline (PBS) or Dulbecco’s Modified Eagle Medium (DMEM). We then palpated males to encourage them to expel fluids. After determining the lavage to be unsuccessful, we palpated males to encourage them to urinate and collected any apparent semen that was mixed with the urine (cloudy string-like substances that were not opaque like uric acid). We collected one sample directly from a male who was courting a female, via aspiration using a syringe in the male’s cloacal opening. We also collected semen samples from females in pairs of tuatara that were observed actively mating, with the male still mounted on top of the female (Fig 2). When the females were captured, the ejaculate was still visible on or near the female’s cloacal opening and was collected using a syringe or microcapillary tube (Fig 3A and 3B) and transferred to an Eppendorf tube. After sample collection, individuals were released at their capture location.

### Recording sperm for velocity analyses

To test the effects of different diluents and cryoprotectants on sperm velocity, we diluted subsamples of semen in either Modified Eagle Medium (MEM), DMEM, or PBS. If sample volume allowed, we also supplemented MEM and PBS storage buffers with glucose. We then pipetted 1ul of sperm diluent mixture and a further 3ul of diluent onto a 20ul Leija slide. We viewed and recorded sperm movement at ambient temperature using a Gigabit Ethernet camera (Basler Scout ACA780-75GC) attached to an Eclipse E200 Nikon microscope, which was set to negative phase and 100x magnification. When samples contained motile sperm, we made recordings and performed velocity analyses using Sperm Class Analyzer software (SCA, Microptic) to capture multiple one second videos from different areas of the slide for each semen sample (Table 1). The treatments selected for each individual’s sample were chosen according to initial results from the previous videos analyzed, and in response to sample volume.

**Table 1:** Tuatara (*Sphenodon punctatus*) sperm sample details.

Male ID	Collection method	Sperm present	Motile
2	Lavage	N	-
4	Lavage	N	-
102	Palpated/urine	Y	Y
102	Aspiration	Y	N
103	Palpated/urine	Y	Y
113	Mating	Y	Y
212	Palpated/urine	Y	Y
213	Palpated/urine	Y	N
240	Palpated/urine	N	-
241	Palpated/urine	N	-
243	Palpated/urine	N	-
244	Palpated/urine	N	-
246	Mating	Y	Y

Characteristics of sperm sample collections from male tuatara (*Sphenodon punctatus*) used in this study. N = No, Y = Yes

### Storage conditions

Following velocity assessments, we subdivided samples containing sperm (regardless of whether it was motile or not) and placed them in different diluents to assess their effects on viability, morphology, and motility of tuatara sperm. Finally, we cryopreserved either undiluted semen or diluted semen samples in one of two cryoprotectants Synth-a-Freeze^™^ or Lake’s Solution. Synth-a-Freeze^™^ contains 10% dimethylsulfoxide (DMSO) buffered with HEPES, a zwitterionic sulfonic acid buffering agent and sodium bicarbonate. Lake’s solution is an avian sperm cryopreservative. Because sperm storage and cryopreservation are largely understudied in reptiles [29], buffers were chosen based on their precedence in both avian and human research. Sperm subsamples were suspended in either modified Eagle’s medium (MEM), MEM plus glucose (MEM + G), DMEM, or PBS. Additionally, we tested two cryoprotectants: Synth-a-Freeze^™^ and Lake’s solution (see corresponding tables for appropriate sample lists). One sample frozen in Lake’s solution was set aside for imaging upon return to the lab.

Finally, an aliquot of semen solution for two individuals with motile sperm (102 and 246) was used for morphology analysis. Each of the samples from these individuals was divided into two conditions; fixed with methanol as smear slides made in the field and the remainder stored in 5% formalin for making smear slides at a later point.

### Morphology

After returning from Takapourewa, we visualized a set of samples cryopreserved with Lake’s solution using scanning electron microscopy (SEM). Briefly, we prepared samples by allowing them to thaw at room temperature before centrifuging at 300 rpm for 1 minute to concentrate sperm. We discarded supernatant and placed a 10 μL drop of sample in the center of an 8 mm glass coverslip. We then allowed sperm to settle for 10 minutes before submerging the coverslip in 1 mL of 2.5% glutaraldehyde in 0.1 M sodium cacodylate buffer for 30 minutes. Next, we pipetted off the buffer and immediately re-submerged coverslips in 1 mL of 0.1 M sodium cacodylate for 10 minutes. After 10 minutes, we pipetted off the sodium cacodylate and submerged coverslips in osmium tetroxide for 30 minutes before removing coverslips and briefly re-treating samples with another 1 mL of 0.1 M sodium cacodylate. We then dehydrated samples with a series of ethanol dilutions progressing from 50% ethanol to three consecutive rinses in 100% ethanol. Finally, we submerged coverslips overnight in 100% acetone. Once fixed, we dried coverslips using a critical point dryer before coating samples with a 5 nm coat of platinum using a Q150T Plus (Quorum). We visualized fixed sperm cells using a JSM-6610 Scanning Electron Microscope (Jeol).

We also viewed pre-prepared methanol-fixed slides and freshly made slides using samples stored in formalin under an Olympus AX50 light microscope at 400x magnification. For each individual and treatment (methanol vs 5% formalin) we took pictures of 20 sperm cells. We measured the total length (μm) of each sperm cell (total *n* = 80) using ImageJ. We calculated the mean and 95% confidence intervals (CIs) for each individual and compared results using a t-test with Holm correction using the R statistical program v4.0.2 [44].

### Viability

**Fig 4:**
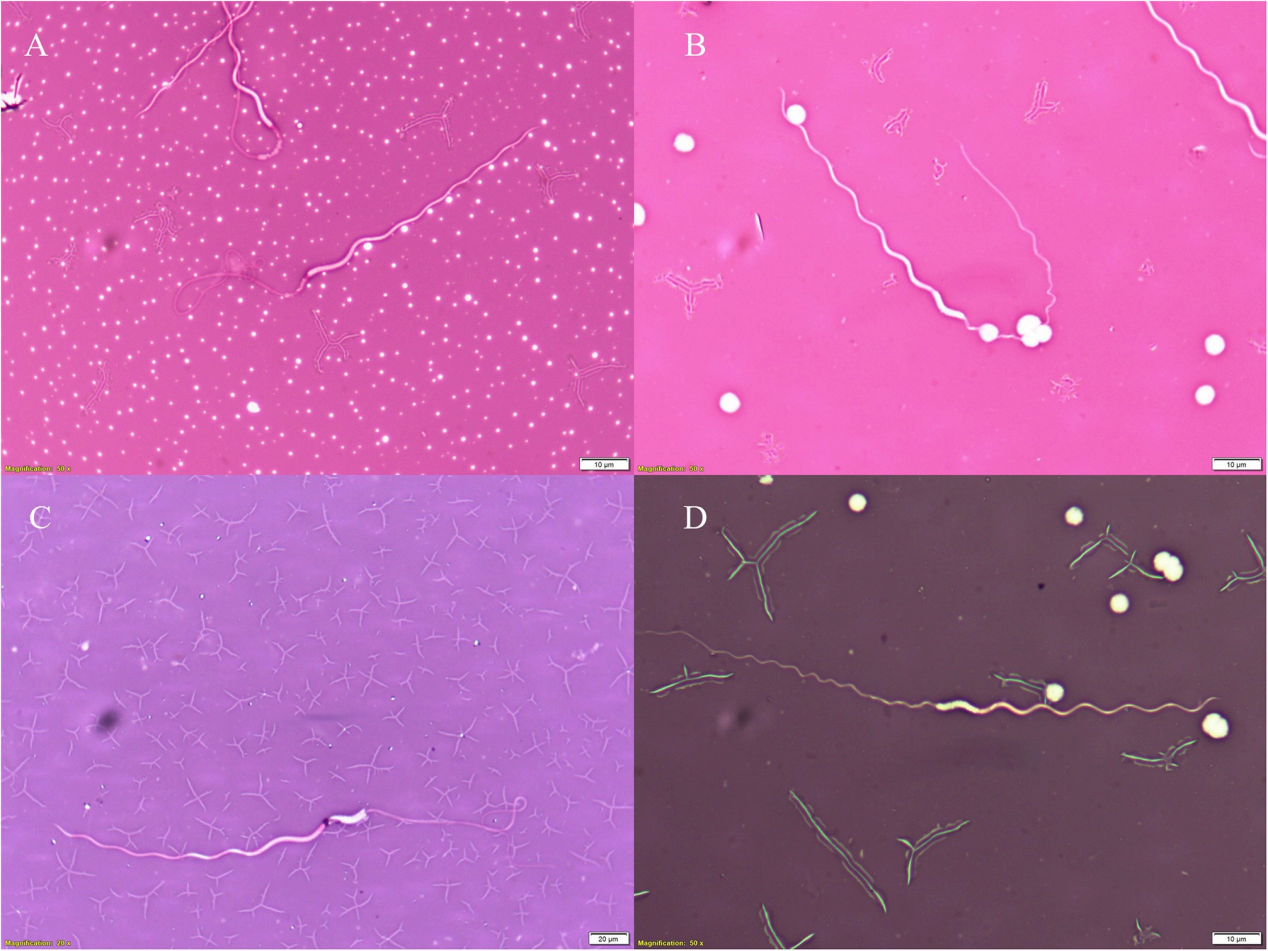
Tuatara (*Sphenodon punctatus)* sperm viability images. (A + C) *Sphenodon punctatus* sperm stained with Eosin Nigrosin to assess viability (membrane integrity). Sperm whose heads are penetrated with stain are non-viable (A + C), while sperm heads that reject stain, and thus appear white, are considered viable (B + D). Various artefacts from the cloaca, in addition to crystals formed by the stain cracking as it dried, are also visible.

Next, we stained sperm samples with an Eosin Nigrosine vital stain exclusion dye, Sperm Vital Stain^™^. We placed a 10μL drop of sperm in the middle of a glass slide and a 10μL drop of Sperm Vital Stain^™^ over the sperm. A second glass slide was placed over the top of the first, and the two slides were pulled apart horizontally to form two slides of sperm stained for viability analyses. Slides were stored in the dark to prevent any light-induced changes in the stain. We assessed sperm viability by counting the total number of sperm and the total number of live sperm on each slide. Samples whose head region was infiltrated with stain were considered unviable (Fig 4A and 4C), while those that excluded stain were considered intact and viable (Fig 4B and 4D). Two researchers (DKO and SKL) performed each count to ensure accuracy. We excluded samples where observer counts varied by ≥ 10%; on this basis we removed two slides. Number of sperm counted, proportion of live cells, and standard deviation were averaged between observers in R.

To assess cell viability (membrane integrity), we first confirmed head region location using DAPI fluorescent staining. Briefly, we prepared samples by allowing them to thaw at room temperature before washing with 40 μL of PBS and centrifuging at 270 rpm for 2 minutes to concentrate the sperm. We discarded the supernatant and spread, with a pipette tip, a 1μL drop of sample in the center of a glass slide. A single drop of DAPI/Prolog Gold with Antifade was placed on the sperm sample and a glass coverslip over the surface. The coverslip was fixed in place with two small dots of clear nail varnish. Sperm were visualized with an Olympus DP80 digital camera attached to an Olympus Fluoview FV3000 confocal microscope.

### Velocity

SCA software provides multiple velocity measures: average path velocity (VAP), curvilinear velocity (VCL), and straight-line velocity (VSL). To assess the impacts of analyzing different subsets of sperm cells per sample, we calculated between-and-within sample variance, as well as intraclass correlation (ICC), of the following groups in R: 1–5 fastest sperm, 1–10 fastest sperm, 1–20 fastest sperm, 1–50 fastest sperm, and 1–100 fastest sperm [45]. Because our sample size of males is small, we treated both sample parameters (male ID and treatment) as our fixed effect and sperm cell VCL (μ × s−1) as the response variable. Based on the results of this analyses, we calculated sperm velocity using a subset of the 5 fastest sperm for each sample. Differences among treatments in individuals 102, 103, and 113 were explored using ANOVAs paired with Tukey post-hoc analyses. Differences between treatments in individual 246 were compared using a pairwise t-test with Holm correction. All analyses were conducted in R.

## Results

### Sample collection

We successfully collected sperm from six adult male tuatara; two sperm collections were obtained from mating pairs, three from urine palpations, and one via cloacal aspiration (Table 1). Of those, only the collections from mating pairs and urine palpations contained motile sperm. Undiluted semen samples collected from mating pairs were small in volume, ranging from 15μL to 28μL. The sperm concentration for male 113 was calculated to be 108 x 10^6^ sperm/mL in a 28uL sample. The total 28 μL semen sample for male 113 therefore contained an estimated 3 x 10^6^ sperm.

### Morphology

**Fig 5:**
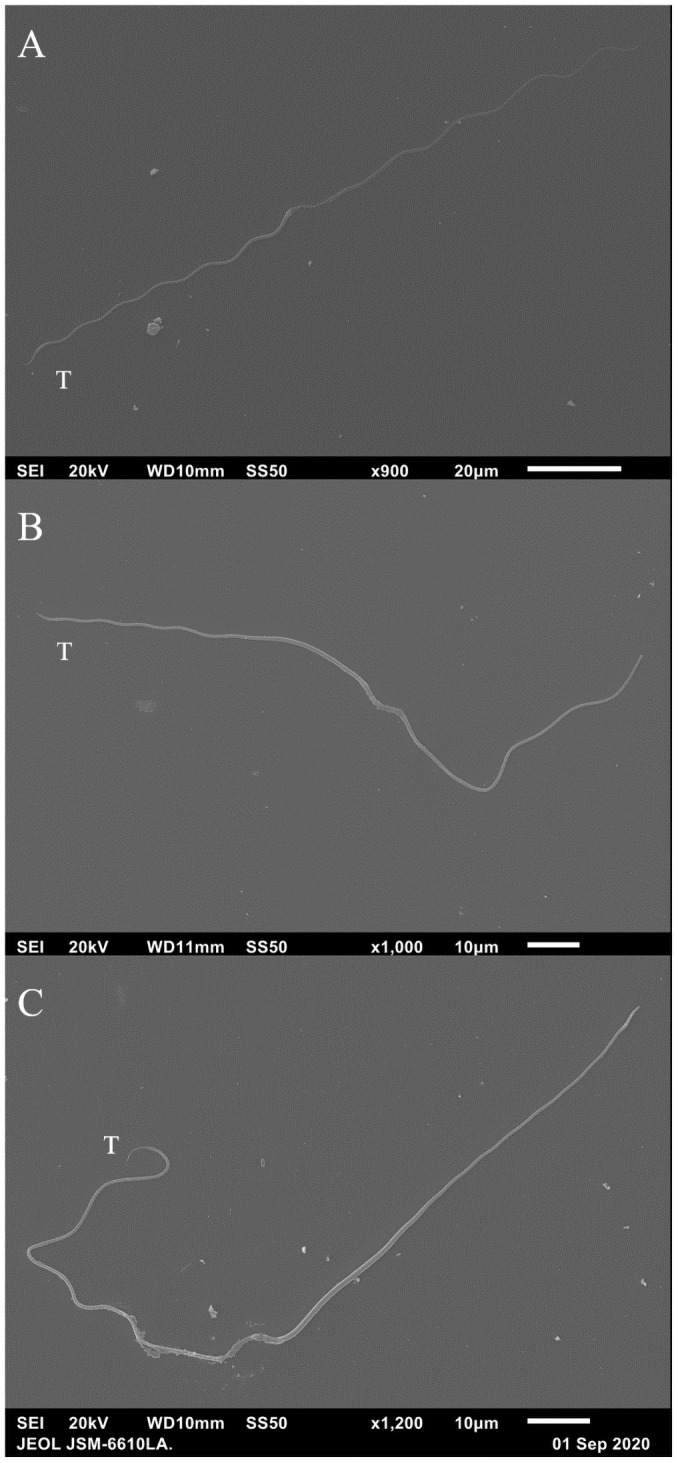
Tuatara (*Sphenodon punctatus*) sperm visualized using SEM. Photographs (A–C) of whole *Sphenodon punctatus* sperm captured using scanning electron microscopy. Sperm had been previously treated with Lake’s solution and flash frozen, before being thawed and photographed. Note the scale difference on the first image. T = tail end.

**Fig 6:**
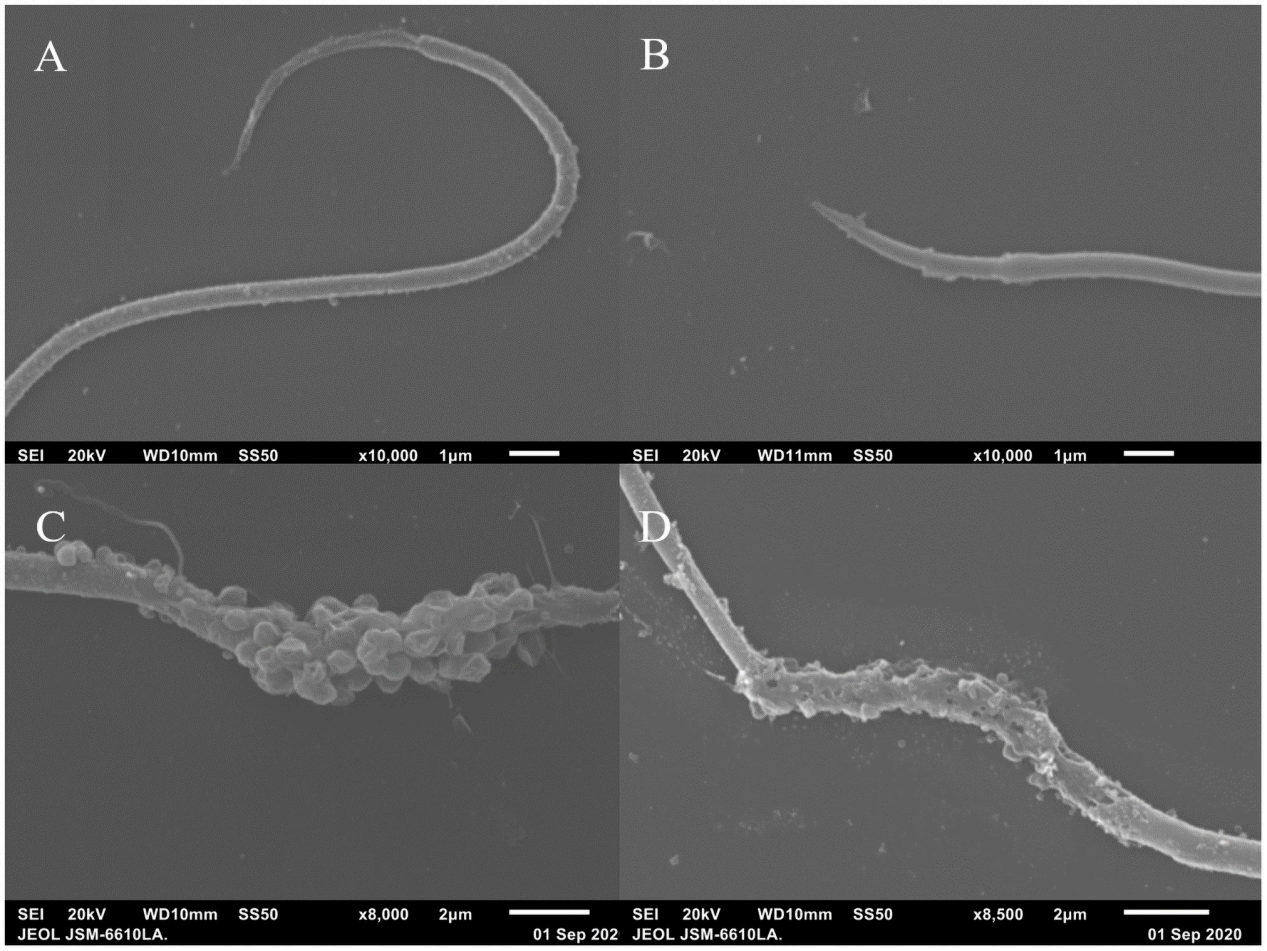
Tuatara sperm tail tips and midpiece damage (*Sphenodon punctatus)*, visualized using SEM. Photographs of *Sphenodon punctatus* sperm treated with Lake’s solution and viewed using scanning electron microscopy, showing (A + B) reduced tail tips and (C + D) different types of midpiece damage, likely due to ineffective cryoprotectant during freezing and thawing.

Sperm frozen in Lake’s solution and viewed under SEM confirmed the filiform, elongated nature of tuatara sperm previously described [18] (Fig 5). Three sections were visible: a thicker head, midpiece, and a thinner tail section. Sperm tails also featured a reduced end piece that began approximately 2.5 μm before the terminus of the cell (Fig 6A and 6B). Midpieces consistently displayed a disrupted external membrane, often with circular structures present (Fig 6C and 6D).

Sperm length varied between individuals, as two males, 102 (mean ± 95% CIS = 126.32 ± 125.03–127.61) and 246 (mean ± 95% CIS = 123.05 ± 121.26–124.83), had significantly different sperm lengths (t = 3.00; p = 0.00369). However, sperm length was not affected by fixatives, as there was no significant difference between the length of sperm from the same individual treated with different fixatives (male 102: t = -1.70, p = 0.0976, male 246: t = -0.55, p = 0.589).

### Viability

The proportion of live sperm cells varied among individuals and treatments (minimum (± sd) = 0.00 ± 0.00, maximum (± sd) = 0.98 ± 0.00). The set of samples with the highest proportions of live sperm were undiluted and obtained via urine palpation during courtship (male 102). The sample set with the next highest proportion of live sperm was again undiluted and was collected from the female’s cloaca during mating (male 246).

The proportion of live sperm cells collected from male 102’s urine during courting was initially high without diluents (0.97 ± 0.03 and 0.98 ± 0.00) but decreased with the addition of MEM + G (0.16 ± 0.06). Similarly, samples from male 103 without added diluents had the highest proportion of live sperm for that individual (0.34 ± 0.01, 0.27 ± 0.01, and 0.27 ± 0.03). The addition of Lake’s cryoprotectant reduced the proportion of live sperm to nearly zero. Male 113’s sperm was exposed to the greatest number of storage method and cryoprotectant treatments. While the number of sperm cells present varied, undiluted semen (0.34 ± 0.00), semen cryopreserved in Synth-a-Freeze^™^ (0.66 ± 0.00 and 0.36 ± 0.06), and sperm diluted with PBS and stored at 4° C for 24 hours (0.30 ± 0.03) had the highest proportions of live sperm. The addition of Lake’s cryoprotectant again led to the least amount of viable sperm present for that individual (Table 2).

**Table 2:** Viability analysis results for tuatara (*Sphenodon punctatus)* sperm.

Male ID	Sample Treatment	*n*	Proportion Live ± sd
*102*			
	Urine collected after courting	247.5	0.97 ± 0.03
	Urine collected after courting	221.0	0.98 ± 0.00
	MEM + G	229.5	0.16 ± 0.06
*103*			
	Lake’s solution	212.5	0.00 ± 0.00
	Lake’s solution	221.0	0.00 ± 0.00
	Urine + Lake’s solution thawed	18.0	0.08 ± 0.00
	Urine + Lake’s solution thawed	19.0	0.00 ± 0.00
	Urine + 24 hours in fridge	65.0	0.34 ± 0.01
	Undiluted semen + 24 hours in fridge	45.5	0.27 ± 0.01
	Undiluted semen + 24 hours in fridge	26.0	0.27 ± 0.03
*113*			
	Undiluted semen	85.5	0.34 ± 0.00
	Undiluted semen + 24 hours in fridge	127.5	0.09 ± 0.01
	Undiluted semen + 24 hours in fridge	214.5	0.07 ± 0.01
	Undiluted semen + Synth-a-Freeze^™^	150.0	0.36 ± 0.06
	Undiluted semen + Synth-a-Freeze^™^	112.0	0.66 ± 0.00
	Synth-a-Freeze^™^+ PBS post-thaw	54.5	0.20 ± 0.00
	Lake’s solution + PBS post-thaw	32.0	0.00 ± 0.00
	Sperm + PBS 24 hours in fridge post-thaw	208.0	0.30 ± 0.03
	Sperm collected from F after mating 1:4 PBS in Lake’s solution	279.5	0.02 ± 0.00
	Sperm collected from F after mating 1:4 PBS in Lake’s solution	231.5	0.03 ± 0.01
*212*			
	Undiluted semen	38.0	0.25 ± 0.06
*213*			
	Palpated urine	25.0	0.10 ± 0.02
	Palpated urine	37.0	0.04 ± 0.02
*246*			
	Undiluted semen from cloaca	205.5	0.83 ± 0.02
	Undiluted semen from cloaca	205.0	0.77 ± 0.01

*Details* of *Sphenodon punctatus* sperm samples stained with Eosin Nigrosin and used for viability analysis, grouped by individual.

*n* = number of sperm counted, MEM = modified Eagle’s medium, G = glucose, PBS = phosphate buffered saline

*n and proportion live values are averages of both researcher’s counts

### Velocity

**Fig 7:**
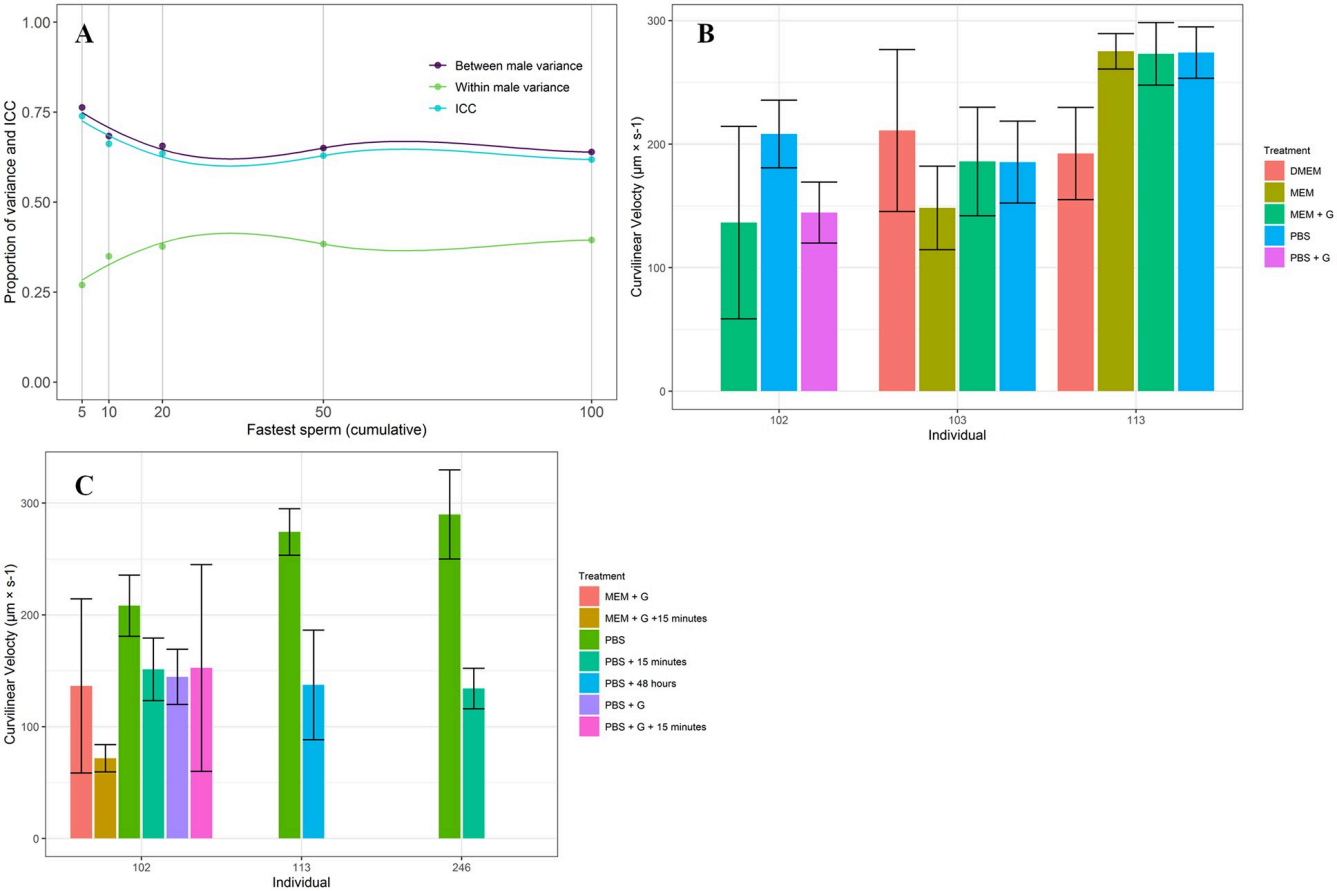
Velocity analyses of tuatara (*Sphenodon punctatus)* sperm. (A) Intraclass correlation coefficients (ICC) and between and within individual variance in *Sphenodon punctatus* sperm speed (curvilinear velocity, μm × s−1) of different groups of fastest sperm cells. The lowest within male variance (0.27), highest between male variance (0.76), and highest ICC (0.74) were all achieved by grouping only the five fastest sperm cells from each sample. (B) Results of movement analysis (curvilinear velocity, VCL) for the five fastest sperm cells per *S*. *punctatus* sample, grouped by individual and treatment. All values presented are in μm × s−1 and bars represent 95% confidence intervals. All samples without the added effect of time are shown. (C) *S*. *punctatus* VCL values (μm × s−1) for all paired samples showing the effect of time on sample velocity. Error bars represent 95% confidence intervals.

The three velocity measures obtained using SCA software were correlated for our samples (VCL vs VAP: r = 0.86, VSL vs VAP: r = 0.72, VCL vs VSL: r = 0.55). While VCL and VSL were not highly correlated, VCL (μm × s−1) is expected to best represent the movement of sperm in the absence of ovarian fluids, and we thus chose to move forward using this velocity measure [46, 47]. Samples had an average of 219 ± 108 sperm cells each (minimum = 105, maximum = 433). The results of our ICC analysis suggest a sample size of the 5 fastest sperm per sample to maximize consistency between sperm cells (ICC = 0.74) and between sample variance (0.76), while minimizing within sample variance (0.27) (Fig 7A). Considering subsets of the 5 fastest sperm per sample, VCL values ranged from 71.71 μm × s−1 (male 102 treated with MEM + G with a 15 minute wait time) to 289.77 μm × s−1 (male 246 treated with PBS).

The impacts of diluent on sperm VCL were inconsistent across samples (Fig 7B and 7C). However, trends are present. Sperm collected from male 103 did not vary in VCL significantly among treatments (5 fastest sperm, p = 0.09). However, males 102, 113, and 246 did. Individual 212 only had one, undiluted sperm sample, whose VCL fell within the range of our other samples (mean VCL = 168.31 μm × s−1).

Sperm collected from male 102 varied significantly in VCL among treatments (p = 0.00) (Fig 7B). Tukey post-hoc analyses showed that while significance was neared on several occasions, it was only found when comparing the fastest and slowest samples. Samples stored in PBS (fastest sample) differed significantly from samples stored in MEM + G with a 15 minute wait time (slowest sample) (p = 0.00). Samples collected from male 113 also varied between treatments (p = 3.29e-08). However, these samples varied significantly between all treatments except MEM and MEM + G (p = 0.99), MEM and PBS (p = 0.99), and PBS and MEM + G (p = 0.99). There was a significant decrease in VCL between PBS and PBS with 48 hours storage at 4°C (Fig 7C). Similarly, male 246’s two samples varied significantly, with the addition of a 15 minute wait time greatly reducing the sperm sample’s VCL (p = 9.3e-06) (Fig 7C).

## Discussion

This study details the first collection and characterization of mature semen samples from wild tuatara (*S*. *punctatus*), the only extant members of the ancient reptile order Rhynchocephalia. Tuatara sperm samples are capable of a very high percentage of viable cells (98%) and have the fastest velocity (VCL) of any reptile sperm yet analyzed. Morphologically, tuatara sperm are filiform and have a thick head, midpiece, and thin tail. Our results also suggest promising directions for tuatara sperm storage, with initial analyses indicating Synth-a-Freeze^™^ may prove to be a suitable long-term cryoprotectant, though more work in this area is needed. This study presents a strong foundation for future ART work and improved conservation of this ancient reptile.

### Collection

Tuatara are unique among reptiles for their lack of intromittent organ [30], which makes sperm collection via methods used in other species difficult. Other sperm collection studies carried out on reptiles often involve euthanizing animals and collecting sperm from the male’s reproductive tract [48–53]. However, tuatara are *taonga* (treasure) to New Zealand’s indigenous Māori people, who retain guardianship over taonga within their *rohe* (territory) [54]. Due to their status as both taonga and a species of conservation importance, euthanizing animals for dissection and sample collection is inappropriate. Further, finding deceased animals in the field is uncommon, and when carcasses are found they are often very degraded. Thus, the ability to collect high quality sperm samples from living, wild tuatara is essential if any ARTs are to be used for this species’ conservation. Our results indicate that the collection of sperm during active mating and from the urine of courting males are both feasible, low-impact ways to acquire viable sperm from tuatara. While we were able to collect sperm via cloacal aspiration of a single individual, this method did not result in motile sperm and is likely not suitable for this species.

### Morphology

While some animal orders, including Aves, display great diversity in sperm morphology, the filiform (thread-like), curved nature of tuatara sperm is common throughout Reptilia [55]. The pointed head region observed in tuatara sperm under SEM is also common in reptiles and some non-passerine birds [55]. Additionally, SEM imaging confirmed the presence of a reduced end piece in tuatara sperm. End pieces consist of only the axoneme and surrounding plasma membrane, lacking the outer dense fibers, fibrous sheath, and mitochondrial sheath present throughout the rest of the sperm tail (Fig 6A and 6B) [56]. We also observed midpiece damage in many sperm cells (Fig 6C and 6D). Disruption of the external membrane has been similarly seen in cases of freeze-thawing sperm when the sperm is stored in ineffective cryoprotectants. We suspect this damage to be due to swelling and disruption of the spherical mitochondria and eventual rupturing of the external membrane [57], but further imaging (including transmission electron microscopy (TEM) and/or freeze-fracture electron microscopy) may help to resolve the nature of this damage. Regardless, these results confirm earlier findings that preservation in Lake’s solution renders tuatara sperm non-viable.

Our results indicate tuatara sperm cells are roughly 125 μm long, which is shorter than previous measurements of testicular sperm in this species (140–144 μm) [18]. This variation in results is likely due to the small sample sizes in both studies and requires further exploration. However, the most common explanation for variation in sperm cell length within a population is a lack of sperm competition, as in passerine birds [58]. The relaxation of post-copulatory competition in a species can lead to diversity in sperm cell morphology. Tuatara are seasonally monogamous, and males on Takapourewa establish territories and mate guard throughout the breeding season [41]. However, low levels of polyandry and polygyny have been found in this population [40]. Additionally, multiple paternity has been observed in tuatara, as well as a period of female sperm storage [38, 40]. Further research is needed to determine the degree of post-copulatory sperm competition in this species, and to determine if the differences in sperm cell length identified between studies is a representation of biological diversity or an artifact of small sample size. Regardless, the total length of tuatara sperm falls within the range of known values in other reptiles, which seem to vary greatly across taxa (e.g. painted turtle, *Chrysemys picta*: 50–55 μm [59] and Wagler’s snake, *Xenodon merremii*: 159.3 ± 3 μm [50]). Further work looking at a larger sample size of tuatara sperm is needed to better link the morphological variation found in sperm length with its donor population.

### Viability

Tuatara sperm samples collected as described have the potential for a very high percentage (98%) of viable sperm cells, but this is variable. Published work on reptile sperm viability (referred to as % intact plasma membrane, IPL) in euthanized Argentine black and white tegus (*Tupinambis merianae*) found initial, untreated sperm IPL values ranging from 94–100% (mean 97.99%) [48], while green ground snake (*Erythrolamprus poecilogyrus sublineatus*) fresh sperm IPL values ranged from 67–92% (mean 80%) [49]. While these results are similar to those found in our samples with the highest viability, variation across individuals and treatments within our study was high. We suspect much of this difference to be related to collection method; samples with the greatest proportion of viable sperm were from urine collected during courting and semen collected from the female’s cloaca immediately following copulation. However, viability of sperm declined over time after collection, with the exception of a single sample. The storage of sperm with high initial viability (male 113) for 24 hours at 4°C led to a significant reduction in viability when stored undiluted rather than diluted in PBS. This is likely because diluents can reduce the adverse effects of reactive oxygen species produced by nonviable sperm.

Of the tested cryoprotectants, the addition of Lake’s solution decreased viability in both individuals’ samples. While commonly used in avian studies, Lake’s solution does not seem a suitable cryoprotectant for tuatara sperm. This result confirms that of the only other published study we were able to find using Lake’s solution as a cryoprotectant on reptile sperm; in the Argentine black and white tegu, no sperm treated with Lake’s solution survived freezing [48]. However, both PBS as a diluent and Synth-a-Freeze^™^ as a cryopreservative showed promise as suitable solutions for the storage of tuatara sperm, as they appeared to maintain cell membrane integrity over time. Further work should examine the impacts of other buffers that have been used with success in reptiles, such as other concentrations of DMSO [53, 60, 61] and TEST-Yolk buffer [26, 48].

### Velocity

Tuatara sperm is 2–4 times faster than that of any previously studied reptile (Wagler’s snake, *X*. *merremii* [50]: maximum VCL ~80 μm × s−1; Argentine boa, *B*. *constrictor occidentalis* [37]: maximum VCL 108.35 μm × s−1; Spectacled caiman, *Caiman crocodilus fuscus* [62]: maximum VCL 57.25 μm × s−1), potentially indicating a novel characteristic of the species that requires further research. While increased sperm speed has been linked with increased promiscuity, and thus sperm competition, in many fish [63, 64] and bird species [45], tuatara on Takapourewa have highly skewed reproduction, with only 25–30% of males typically mating each season [40]. The rapid VCL of tuatara sperm analyzed in this study warrants further investigation into both the pre- and post-copulatory selection pressures of this population and the potential of increased VCL to function as an adaptation to the lack of male copulatory organ. In birds lacking intromittent organs, which also mate via cloacal apposition, there are several traits that potentially serve to improve the chances of successful sperm deposition. For example, the Japanese quail (*Coturnix japonica*) produces a foam from its cloaca, which is thought to help facilitate insemination, prevent sperm from exiting the female reproductive tract, and thus aid eventual egg fertilization [65]. The rapid VCL found in tuatara sperm, in combination with the prolonged mounting and side-to-side movement of the male on top of the female following copulation, may serve a similar purpose.

Finally, our velocity analyses indicate that a 15 minute wait period at ambient temperature results in a significant decrease in sperm VCL (Fig 7C). Samples stored in PBS, DMEM, and MEM all offered promising results that require further exploration in a larger sample pool to help clarify patterns created by differences in individual sperm quality. While the importance of motile sperm to successful artificial insemination after cryopreservation has not, to our knowledge, been investigated in reptiles, it has been extensively studied in mammals. Retention of high concentrations of motile sperm after thawing of frozen samples is consistently linked with successful pregnancies after artificial insemination in humans [66, 67], making the identification of a cryoprotectant and storage temperature regime that will result in the maintenance of motile tuatara sperm critical to future use of ARTs for this species.

## Conclusion

This research provides the first characterization of mature sperm from the archaic reptile tuatara. While further work is needed to observe trends at a larger scale, we have confirmed that sperm collection from mating pairs and urine collected from courting males are both safe and viable methods for the collection of living, motile sperm from wild populations of tuatara. We also present initial characterization of sperm morphology, viability, and velocity—which is the fastest yet analyzed among reptiles. This result could indicate a novel adaptation of this unique reptile intended to improve the likelihood of successful sperm deposition and eventual egg fertilization in the absence of a male intromittent organ. This research not only adds foundational information to our understanding of tuatara, but also provides important context for sperm characteristics within Reptilia, a taxon in which reproductive characteristics are understudied, despite one in five reptile species being threatened with extinction [68, 69]. Finally, we have taken the first steps in parsing out a suitable storage method for the eventual cryopreservation of tuatara gametes. This work has important, practical applications towards the development of conservation breeding techniques and improved genetic management of this unique reptile, helping to ensure the persistence of the last of the Rhynchocephalians.
